# Inferring Networks of Interdependent Labor Skills to Illuminate Urban Economic Structure

**DOI:** 10.3390/e22101078

**Published:** 2020-09-25

**Authors:** Shade T. Shutters, Keith Waters

**Affiliations:** 1 School of Complex Adaptive Systems, Arizona State University, Tempe, AZ 85282, USA; 2 Schar School of Policy and Government, George Mason University, Fairfax, VA 22201, USA; kwaters2@gmu.edu

**Keywords:** urban science, regional science, cities, workforce, resilience, Panarchy, information theory, interdependence, co-occurrence

## Abstract

Cities are among the best examples of complex systems. The adaptive components of a city, such as its people, firms, institutions, and physical structures, form intricate and often non-intuitive interdependencies with one another. These interdependencies can be quantified and represented as links of a network that give visibility to otherwise cryptic structural elements of urban systems. Here, we use aspects of information theory to elucidate the interdependence network among labor skills, illuminating parts of the hidden economic structure of cities. Using pairwise interdependencies we compute an aggregate, skills-based measure of system “tightness” of a city’s labor force, capturing the degree of integration or internal connectedness of a city’s economy. We find that urban economies with higher tightness tend to be more productive in terms of higher GDP per capita. However, related work has shown that cities with higher system tightness are also more negatively affected by shocks. Thus, our skills-based metric may offer additional insights into a city’s resilience. Finally, we demonstrate how viewing the web of interdependent skills as a weighted network can lead to additional insights about cities and their economies.

## 1. Introduction

Cities are the driving force behind many of humanity’s problems today [1,2]. Yet they are also a key source of potential solutions, and thus their welfare and sustainability are critical for humanity’s future [3,4]. Now home to over half of all humans on earth, cities are increasingly susceptible to the effects of shocks such as economic recessions, global pandemics, and natural disasters [5]. Shocks often damage parts of a city’s structure that may require long periods of time and substantial resources to repair. Thus, it is imperative that we develop a deeper understanding of urban structures, how those structures respond to shocks, and how policy makers might alter those structures to enhance a city’s resilience [6,7].

But what does it mean to say a city has structure? Physical attributes, such as the built environment and natural features, are more obvious structural components of cities. Yet many less obvious structures exist. For instance, local norms, customs, and laws can be viewed as part of a city’s institutional structure [8]. Similarly, distributions of employment across industries that remain relatively stable over time can be viewed as part of a city’s economic structure. It is these less obvious structural components of cities that we seek to understand, particularly how they respond to different shocks and how they contribute to the resilience of cities.

We adopt the view that urban systems are sublime examples of complex adaptive systems. We take this to be more than mere metaphor, as cities are systems of adaptive entities interacting both with each other and with their surrounding environment. These interactions are governed by complex networks that are themselves adaptive, dynamic, and interconnected with each other [9]. Cities are thus complex networks of networks [10,11] and advancing knowledge of how cities function and respond to shocks requires a deeper understanding of the numerous interconnected networks both within and between cities. While networks such as roadways, power conduits, and resource flow patterns, are easily observable, we focus here on those interaction networks that are more cryptic and typically not revealed through direct observation.

We take temporally stable distributions of some city attribute to be one type of urban structure, focusing on economic structures and how they might respond to shocks. In addition to a city’s industrial structure mentioned previously, another relatively stable distribution is a city’s proportion of workers in each labor occupation. We take this distribution to be a key element of a city’s labor structure and use elements of information theory to elucidate the interaction network among a city’s prevalent labor skills. We further quantify global attributes of this network to understand how it relates to economic performance and how it may anticipate a city’s response to economic shocks.

We implement an emerging technique that uses distributions of workers by occupation to understand structural elements of an urban economy [12,13,14,15]. One use of this technique has been to measure the level of interconnectedness within an urban economy’s workforce, revealing that interconnectedness, or economic tightness, is positively correlated with more severe declines in economic performance following a shock [16]. This result concurs with the so-called Panarchy theory of complex adaptive systems [17,18], which asserts that systems with higher internal connectedness are more efficient, but also more brittle and vulnerable to disruption. Thus, economic tightness is intimately linked to fragility and resilience, and its importance arises from its potential to help anticipate impacts of system shocks, particularly economic shocks.

Here, we apply this methodology to distributions, not of labor occupations, but of labor skills to calculate a novel metric of regional economic tightness. Skills are typically viewed as more relevant attributes for relating labor to economic output but historically have been more difficult to quantify at a regional scale than occupational counts. Consequently, occupations are frequently used as a convenient proxy for measuring labor skills. However, researchers are now beginning to elucidate economic structures directly from labor skills data [19]. Adopting this framework, we compare a city’s skills-based economic tightness to measures of productivity, to response to an economic shock, and to previously used calculations of economic tightness based on occupations.

## 2. Materials and Methods

### 2.1. Data and Sources

Our geographical units of analysis are the 395 metropolitan statistical areas (MSAs) of the U.S. for which 2018 occupational employment data are available. MSAs are defined as a core county, or counties, containing an urbanized area with a population of at least 50,000, plus adjacent counties having a high degree of social and economic integration, primarily measured as commuting ties [20]. Thus, MSAs are considered unified labor markets and encompass geographical areas of high economic cohesion.

We merge two publicly available data sets to calculate an MSA’s aggregate level of each labor skill. The first is the Occupational Employment Statistics (OES) published annually by the U.S. Bureau of Labor Statistics [21]. OES data include an annual estimate of the number of workers in each occupation in each MSA. We use the May 2018 OES dataset, which was the latest version available at the time of our study.

We note here one idiosyncrasy of the OES dataset. While the Bureau of Labor Statistics describes its OES data as covering MSAs, it actually uses an alternative geographical unit within the six New England states. For those states, the BLS aggregates employment not to MSAs but to an alternate federal statistical unit known as New England City and Town Areas, or NECTAs, which are not based on counties. Though MSAs and NECTAs often share the same description, e.g., greater Boston, they do not encompass the same geographical areas. This variance does not materially affect our analysis, but it does create comparability issues with data from other sources, such as the U.S. Bureau of Economic Analysis, that use standard MSAs for New England data. Thus, researchers that may use our methodology in conjunction with other data sources should be aware of this issue.

The second dataset is version 24.2 of the Occupational Information Network or O*Net [22]. O*Net decomposes U.S. occupations into several hundred attributes, representing the typical skills, activities, knowledge, and abilities required or used by workers in each labor occupation. This decomposition is done using two distinct methods. The first decomposes occupations into 258 so-called elements, examples of which include “Oral Comprehension”, “Management of Material Resources”, and “Design.” Elements are assigned a numeric value for each occupation based on the element’s level of importance to a worker in that occupation. A single element can have values for multiple dimensions of the same occupation, e.g., importance, relevance, frequency, extent, or level. Thus, for a given occupation, a single element, such as Oral Comprehension, may have different values for importance, level required, frequency of use, extent, etc. In this study, we use the level of an element, for which 161 elements have a value. We also performed our analysis using the importance values of elements but found no appreciable differences from results using level and thus, for conciseness, do not report on those supplemental results.

The second O*Net method decomposes occupations into 322 individual work activities (IWAs). Unlike elements, IWAs are not assigned a value but are identified as either present or absent in each occupation. Examples of IWAs include “Collect information about patients and clients”, “Monitor environmental conditions”, and “Design databases.” Hereafter, we use the term “skills” to refer collectively to all the various O*Net elements and IWAs.

In our analysis, we correlate our urban structural metrics with urban economic performance, measured as gross domestic product (GDP) per capita. We calculate per capita GDP using 2018 MSA-level aggregate GDP and population, both extracted from the U.S. Bureau of Economic Analysis [23].

### 2.2. Quantifying Interdependence

To quantify an aggregate measure of interdependence characterizing each MSA’s economy, we first transform each MSA’s distribution of occupational employment (from OES data) into a distribution of skills intensity. We do this by mapping OES occupation codes to their O*Net counterpart (see Supplemental Materials) and then, for each MSA, multiplying the number of workers in an occupation by the occupation-specific value of each skill, or, in the case of IWAs, the number of workers whose occupation possesses the given IWA:

(1) $$s_{i,m}=\underset{o}{\sum }l_{i,o}w_{o,m}$$

where *w* is the number of workers employed in occupation *o* in MSA *m*, and *l* is the quantified level of skill *i* in occupation *o*. When using elements *l* ∈ [1, 5], and when using IWAs *l* ∈ {0, 1}. The resulting *s* is an aggregate measure of each skill’s presence in an MSA’s labor pool.

We next determine whether each skill is present or absent in each MSA. While it is likely that every skill exists in every MSA at some nominal level, we apply a threshold to determine presence or absence for our purposes. We do this by applying the widely used metric of location quotient (*LQ*):

(2) $$LQ_{i,m}=\frac{\left(\;s_{i,m}/\sum_{i}s_{i,m}\right)}{\left(\sum_{m}s_{i,m}/\sum_{m}\sum_{i}s_{i,m}\right)}\;.$$

In short, *LQ* is the ratio of the local concentration of a skill compared to the national concentration of that skill. Following [12], we take a skill *i* to be present in MSA *m* if *LQ_i,m_* ≥ 1 and absent if *LQ_i,m_* < 1. This results in an MSA × skill matrix of presence–absence data.

This matrix is then used to calculate the probability that a skill is present in a randomly selected MSA as well as the probability that a given pair of skills will co-occur in a random MSA. Using a variant of mutual information defined in [12], we next quantify the interdependence *x* between any two skills *i* and *j* as:

(3) $$x_{i,j}=\frac{P\left[LQ_{i,m}>1,LQ_{j,m}>1\right]}{P\left[LQ_{i,m^{\prime }}>1\right]P\left[LQ_{j,m^{\prime \prime }}>1\right]}-1,$$

where *m*, *m′*, and *m′′* denote randomly selected MSAs. Thus, interdependence is the conditional probability of two skills occurring together compared to the product of their marginal probabilities. The result is a skill × skill matrix of interdependence values. Two skills that co-occur in MSAs more frequently than expected by chance will have a positive interdependence value, while two skills that co-occur less frequently than expected will have negative interdependence. We take this matrix of interdependence values to be the adjacency matrix of a complete weighted network describing how worker skills interact across the entire U.S. Note that *x_i,j_* = *x_j,i_* meaning the adjacency matrix is symmetric and thus the network is undirected.

We then implement the methodology of [16] to calculate an aggregate measure of MSA interdependence, or tightness. This method dictates that we first assign an MSA-specific weight *L* to each MSA’s pair of present skills using the pair-wise interdependence *x* and weighting by the local proportions of each:

(4) $$L_{i,j,m}=\frac{\left(s_{i,m}+s_{j,m}\right)x_{i,j}}{2\sum_{i}s_{i,m}}$$

where *i* and *j* are skills both present in MSA *m*. We then average *L* across the total number of links in an MSA’s skills subnetwork to produce a skills-based tightness metric:

(5) $$T_{m}=\frac{2}{p_{m}\left(p_{m}-1\right)}\overset{p_{m}}{\underset{i<j}{\sum }}L_{i,j,m}$$

where *i* and *j* are both present in MSA *m* and *p_m_* is the total number of skills present in MSA *m*. Note that averaging the weight *L* across all links in an MSA’s subnetwork is equivalent to the generalized network density of that subnetwork [24].

Finally, because tightness is a dimensionless measure based on an arbitrary scale of skill level, we normalize tightness values as a z-score with mean = 0 and standard deviation = 1.

## 3. Results and Discussion

### 3.1. Skills and Interdependence

**Figure 1:**
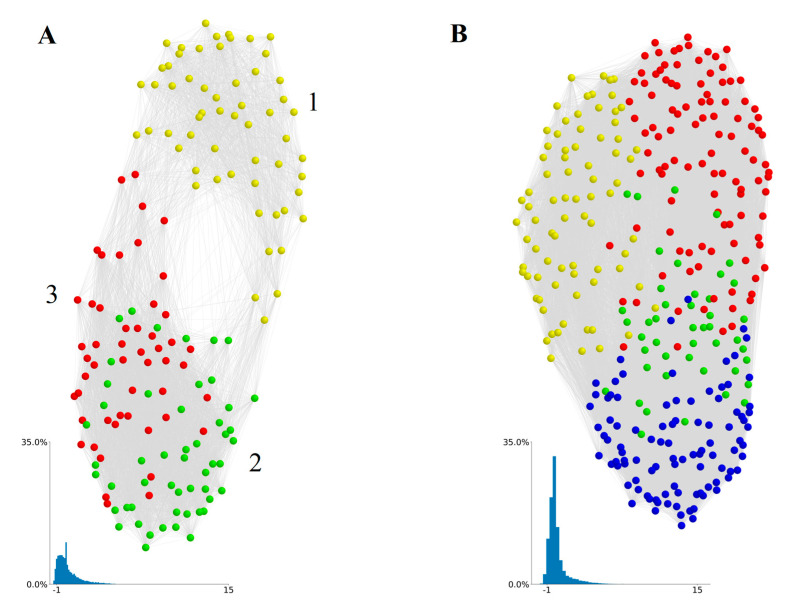
Skill interdependence networks constructed from O*Net (**A**) elements and (**B**) individual work activities (IWAs). Nodes are skills and proximity between skills is a function of pairwise interdependencies. Non-normalized edge weights are displayed. All edges greater than zero are displayed. Node colors are determined using the Kamada–Kawai community detection algorithm. In the element-based skills network (**A**), yellow nodes = Sensory-physical skills, green nodes = Socio-Cognitive: Technical skills, and red nodes = Socio-Cognitive: General skills. In the IWA-based skills network (**B**), four communities emerged (colored differently) but they form unintuitive groupings of skills. Insets show distribution of normalized link weights in each network.

To calculate the economic tightness of MSAs, we first calculated an interdependence value for each possible pair of skills, including 12,880 element pairs and 51,681 IWA pairs. The distributions of values using each skill type are shown as insets in Figure 1. We take the full skill × skill matrix of interdependence values to be the adjacency matrix of a complete and weighted national-level interdependence network, in which nodes are the various labor skills and the weights are pair-wise interdependence values (Figure 1). Networks were created using the Kamada–Kawai algorithm, which produces evenly spaced nodes for relatively small networks and minimizes the number of edge crossings [25,26]. Because interdependence captures the degree to which skills tend to co-occur across MSAs, two skills that are highly interdependent tend to be near to each other in our network, while pairs with a low or negative interdependence tend to be farther apart. Negative interdependence indicates that if one skill of a pair is present in a city, the other tends not to be present. Examples of skill pairs with high interdependence and low interdependence are shown in Table 1.

While the IWA-based network (Figure 1B) shows little global structure, the element-based network (Figure 1A) displays a double-lobe structure noted in a comparable study on O*Net skills [19], a phenomenon the authors refer to as skills polarization. However, the authors of [19] constructed their network’s interdependencies using the location quotient of skills across occupations, while we have constructed ours using an aggregate measure of skills across MSAs. Thus, this polarization exhibited by O*Net elements is seemingly robust to a variety of construction methodologies.

To uncover communities within the skills interdependence network, we remove edge with weights less than zero and use the Louvain community detection algorithm (LCD) with single refinement [27]. The LCD algorithm iteratively optimizes modularity by measuring the relative density within and between communities. The exclusion of edge weights lower than zero is justified on the grounds that such weights represent a type of repulsion between skills.

In the element-derived network, we find a similar polarization of skills with LCD identifying three communities (numbered in Figure 1A). Region 1 (yellow nodes) corresponds closely to what [19] refers to as a “sensory-physical” skills cluster, which includes elements such as Finger Dexterity, Spatial Orientation, and Production and Processing. Regions 2 (green nodes) and 3 (red nodes) correspond closely to what [19] refers to as the “socio-cognitive: technical” and “socio-cognitive: general” skills clusters, respectively, containing elements such as Medicine and Dentistry, Fine Arts, Economics and Accounting, and Technology Design. We refer to regions 2 and 3 collectively as the socio-cognitive lobe of our network.

On the other hand, the LCD algorithms identified four skill communities in the IWA network (Figure 1B) but they do not correspond to obvious or intuitive groupings of skills. Members of both element and IWA skills communities are presented in the Supplemental Materials. One possibility for less meaningful structure with IWAs is that, unlike elements, IWAs are not quantified, but are simply present or absent in an occupation. Thus, while an IWA might be present in two occupations, it may be critical to one occupation and only marginally important to the other. Yet this difference in importance is not captured using IWAs.

**Figure 2:**
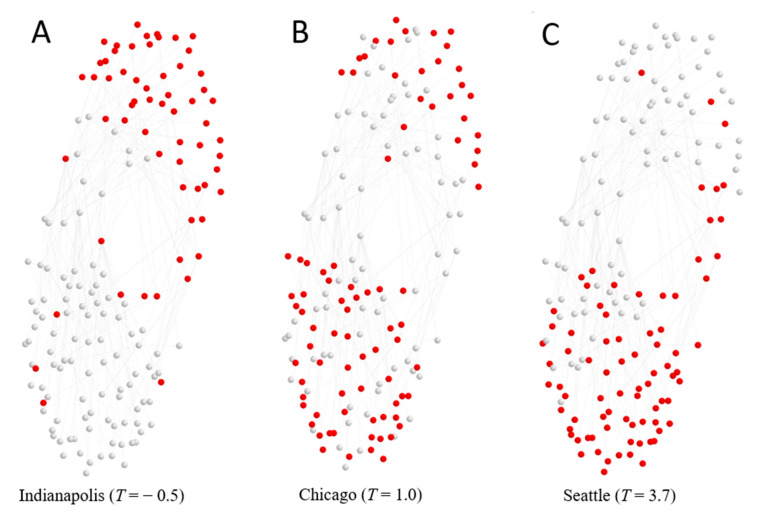
Locations of three representative metropolitan statistical areas (MSAs) within the national skills (elements) interdependence network. (**A**) Seattle is located almost exclusively within the socio-cognitive cluster of skills, while (**B**) Chicago is more balanced across lobes and (**C**) Indianapolis is largely within the sensory-physical cluster. Normalized tightness scores of each city are shown in parenthesis.

Because a subset of possible skills is present in each MSA, an MSA can be viewed as a subnetwork of the national-level interdependence network. We take this subnetwork to be the “location” of a given MSA within a national map. Examples of three such subnetworks, representing cities with low, medium, and high values of tightness, are shown in Figure 2. Among these example cities, as tightness increases, a city’s location shifts from the sensory-physical lobe of the skills network to the socio-cognitive lobe.

### 3.2. MSAs and Tightness

**Figure 3:**
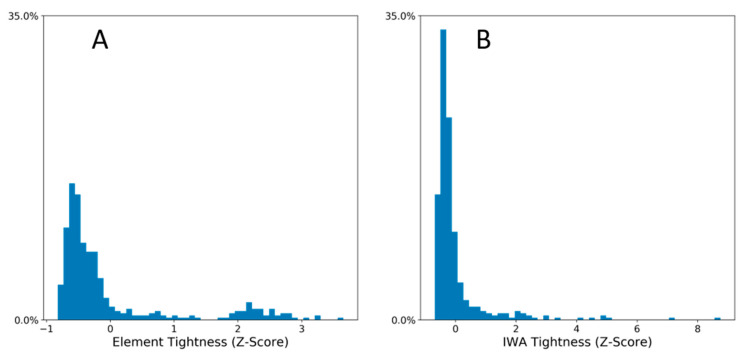
Frequency distribution of economic tightness *T* across MSAs. (**A**) *T* derived using O*Net elements and (**B**) O*Net individual work activities (IWAs). Distributions based on 2018 occupational employment data, *N* = 395 MSAs in both panels.

Interdependence values of skill pairs become the basis of an aggregate metric of economic integration or connectedness that we refer to as tightness *T*. Using both elements and IWAs as skills, we calculated *T* for each of the 395 MSAs in our study. Distributions of *T* using each skill type are shown in Figure 3. Note that the distribution of *T* based on elements is clearly bimodal, with a small cluster of MSAs having significantly higher tightness than the remaining MSAs. A list of MSAs having the highest and lowest tightness values are presented in Table 2 (where *T* is based on IWAs as skills).

### 3.3. Spatial Distribution and Autocorrelation of Tightness

**Figure 4:**
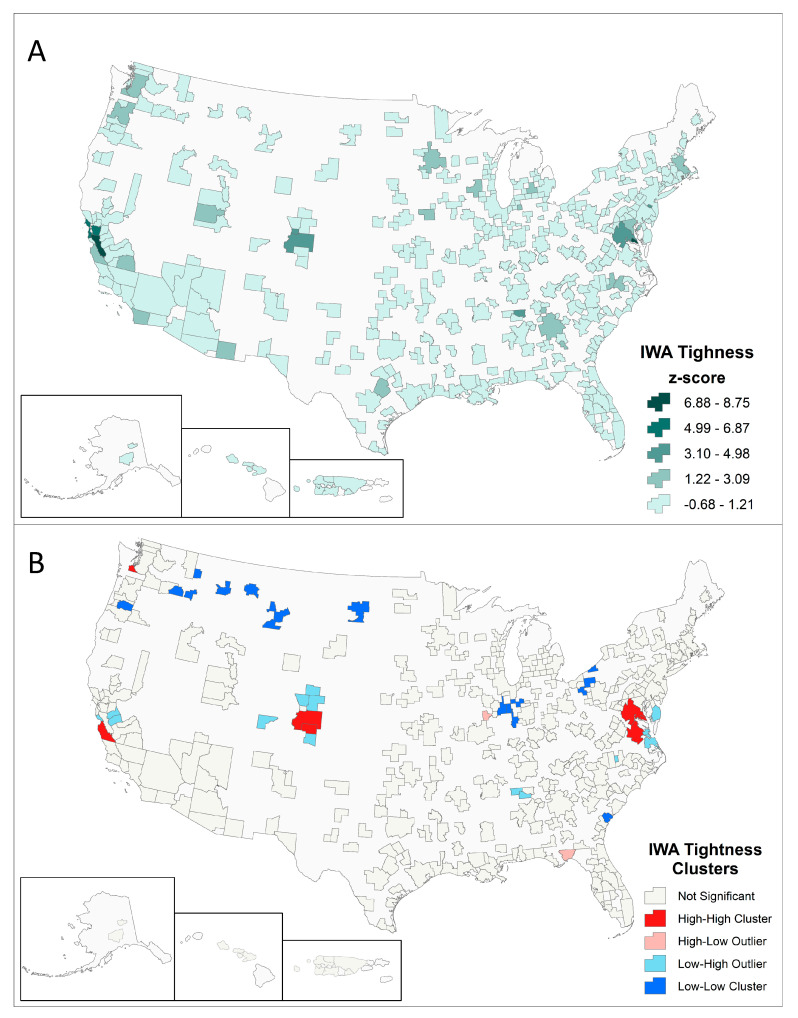
Spatial distribution and autocorrelation of MSA tightness values. (**A**) IWA Tightness and (**B**) Anselin’s Local Moran’s I (LISA). Clusters significant at a confidence level of 0.05 using *k*-nearest neighbors, *k* = 4.

MSAs with high tightness are relatively well-dispersed spatially (Figure 4A). While MSAs with high tightness are prominent along U.S. coastal regions, several exist inland, such as Denver, Minneapolis, and Huntsville. We apply Anselin’s Local Moran’s I [28], a measure of spatial association, to identify statistically significant clusters of MSAs with both high tightness (High–High clusters) and low tightness (Low–Low clusters) (Figure 4B). Two other cluster types, High–Low and Low–High, are typically MSAs with low tightness adjacent to MSAs with high tightness. We find four High–High clusters—Olympia, San Francisco, Denver, and Washington, DC. Low–Low clusters are more numerous and are concentrated around the Great Lakes and in the Northern Great Plains.

One possible reason for these High–High clusters is that they result from spillovers of tightly connected economic activities to surrounding MSAs. For example, if a highly integrated MSA increases rents, interdependent economic activity may move to nearby MSAs that are cheaper but spatially proximate. This could both displace existing economic activities and increase the tightness of such nearby MSAs.

### 3.4. Tightness, Productivity, and Economic Shocks

To analyze the relationship between tightness and economic productivity, we compared an MSA’s skills-based tightness to both its per capita GDP and per capita personal income. Results show that per capita GDP is significantly and positively correlated with tightness, using both elements (R = 0.40, *p* < 0.001) and IWAs (R = 0.44, *p* < 0.001). The relationship between tightness and per capita GDP remains positive and significant when controlling for MSA population (see Supplemental Material). Likewise, per capita personal income is significantly and positively correlated with element-based tightness (R = 0.43, *p* < 0.001) and IWA-based tightness (R = 0.40, *p* < 0.001). These results concur with previous work that calculated MSA tightness using the interdependence values between occupations, as opposed to skills [16]. Thus, both our skills-based tightness and the previously published occupation-based tightness are associated with higher economic productivity in general.

However, these two measures differ in their relationship to a city’s response to a shock. While [16] found that occupation-based tightness is negatively correlated with a city’s change in GDP during the 2007–2009 recession, our study finds that skills-based tightness (elements, 2006 data and 2006 MSA definitions) is positively correlated with change in GDP during the recession (R = 0.12, *p* < 0.05). Thus, occupation-based tightness and skills-based tightness measures have opposite relationships to an urban economy’s response to shocks.

The finding that cities with higher occupation-based tightness had larger percentage drops in productivity following a shock is consistent with the Panarchy theory of resilience, which asserts that as systems increase in connectedness, they become more brittle and fragile. However, the opposite relationship of this study’s skills-based tightness is more difficult to explain. One possible reason is that, following a shock, skills are more transferable to new occupations, while occupations are not as easily transferable to new industries. Thus, even if a city’s GDP declines after a recession, some workers maintain their existing jobs by utilizing their skill sets in a new capacity, but not by changing occupations.

Another plausible explanation relies on the ecological concepts of niches and competitive exclusion [29], where occupations act as species and an urban economy as an ecosystem. Under this framework, occupation pairs with low interdependence share the same economic role, or niche, which can only be occupied by one occupation at any given time in a city. In this case, the occupations of this type of pair competitively exclude one another and, because they perform a similar economic function, are likely to have similar skill sets. Thus, even when two occupations have low interdependence, some of the skills they share are likely to have high interdependence. For example, the occupations Industrial Engineer and Petroleum Engineer have a strong negative interdependence (*x* = −0.69), meaning they rarely appear in the same city together. Yet both occupations have high O*Net values (>4.0 on a scale of 1 to 5) for skills like Systems Analysis, Programming, and Complex Problem Solving, all of which are highly interdependent with each other (*x* > 4.0).

### 3.5. GDP vs. Resilience: A Policy Tradeoff Frontier

**Figure 5:**
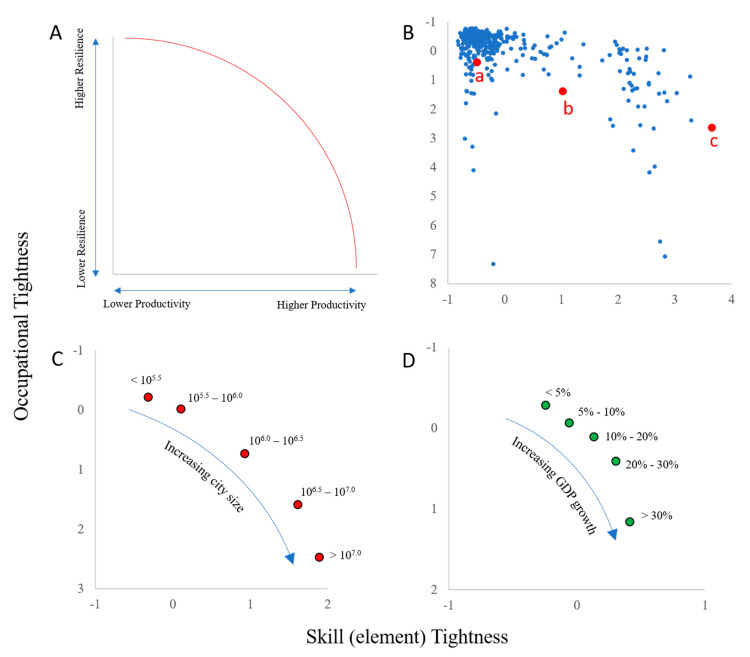
A conceptualized tradeoff between occupation-based and skills-based tightness. (**A**) stylized tradeoff model with the red arc representing a Pareto frontier indicative of a tradeoff. (**B**) actual data for 395 MSAs using 2018 labor data. MSAs labeled a, b and c correspond to example city networks in Figure 2. (**C**) plots the tightness centroids of cities grouped into five bins based on population size (bin sizes shown next to points), while (**D**) does the same for cities grouped into bins based on 5-year GDP growth (growth bins shown next to points). Tightness measures have been normalized as z-scores with the *y*-axis inverted in all cases.

While we find that skills-based tightness and occupation-based tightness have opposite correlations with GDP growth following a shock, they are nevertheless positively correlated with each other (2018 data, R = 0.54 for elements, R = 0.78 for IWAs, *p* < 0.001 for both), giving rise to a tradeoff between labor structures that promote resilience and those that promote higher economic productivity. This tradeoff can be viewed as a Pareto frontier in a plane plotting skills-based tightness against occupation-based tightness, as conceptualized in Figure 5A. When 2018 tightness metrics for the 395 MSAs of our study are plotted in such a tightness plane, an approximation of a Pareto frontier does seemingly emerge (Figure 5B).

To explore the role of a city’s size on its location in this plane, we group MSAs into bins based on log(population) and plot the centroid of each group in the same plane. The result (Figure 5C) reveals that as cities increase in size, they tend to move along a Pareto frontier from low skill tightness and low occupation tightness toward higher skill and occupation tightness. A similar trajectory is revealed when cities are grouped by 5-year GDP growth (Figure 5D). These results suggest that as cities develop and grow, they follow a generalized trajectory becoming both more economically productive and more susceptible to detrimental effects of economic shocks.

This conceptual model suggests that policy makers, to the extent that they can control a city’s portfolio of skills and occupations, can influence their region’s position along this trade-off frontier, fostering growth in unrelated occupations if they are more concerned with resilience, or fostering growth in interdependent skills if they are more concerned with GDP. However, Figure 5C also suggests that because a city’s position is related to population size, policy makers are likely constrained in their ability to influence their city’s position.

### 3.6. IWAs versus Elements

As pointed out in the methods, O*Net creates two sets of data, elements and IWAs, each of which can be used to create a vector of attributes associated with each U.S. occupation. One goal of this study was to understand how results using labor decomposition might differ using these two alternatives. Thus, throughout this study we have applied a consistent methodology using both datasets.

In general, we find that the elements provide more meaningful results than IWAs for the purposes of this study. For instance, in Figure 1 elements lead to three intuitive clusters of skills and a multi-lobe structure of its interdependence network, while the IWA network displays no lobes and four skills clusters that are difficult to interpret. Similarly, in Figure 3, tightness values derived from elements exhibit a multimodal distribution, indicative of complex systems with multiple possible states [30], while the distribution derived with IWAs exhibits no such mutlimodality.

While elements are assigned a magnitude for each occupation, IWAs are assigned as either present or absent on an occupation. It is likely that this quantified magnitude leads to the richer outcomes found with elements. Thus, researchers seeking to decompose occupations for analytic purposes may wish to consider these points.

## Figures and Tables

**Table 1:** Highest and lowest ranked IWA pairs based on interdependence ***x_i,j_***.

Rank	*i*	*j*	*x_i,j_*
1	Study details of artistic productions	Present arts or entertainment performances	15.5
2	Study details of artistic productions	Alter audio or video recordings	13.1
3	Alter audio or video recordings	Present arts or entertainment performances	11.7
4	Consult legal materials or public records	Discuss legal matters with clients, disputants, or legal professionals or staff	9.9
5	Study details of artistic productions	Develop news, entertainment, or artistic content	9.8
55,108	Plan events or programs	Hunt animals	−1.6
55,109	Clean tools, equipment, facilities, or work areas	Direct scientific or technical activities	−1.7
55,110	Analyze scientific or applied data using mathematical principles	Clean tools, equipment, facilities, or work areas	−1.7
55,111	Hunt animals	Prepare proposals or grant applications	−1.8
55,112	Evaluate scholarly work	Hunt animals	−1.8

**Table 2:** Highest and lowest ranked MSA tightness values *T*, using IWAs as skills.

Rank	Metropolitan Statistical Area (MSA)	*T* *
1	San Jose–Sunnyvale–Santa Clara, CA (41,940)	8.75
2	California–Lexington Park, MD (15,680)	7.05
3	San Francisco–Oakland–Berkeley, CA (41,860)	5.09
4	Boulder, CO (14,500)	4.89
5	Huntsville, AL (26,620)	4.84
391	Kennewick–Richland, WA (28,420)	−0.65
392	Montgomery, AL (33,860)	−0.65
393	New Bern, NC (35,100)	−0.65
394	Bellingham, WA (13,380)	−0.66
395	Knoxville, TN (28,940)	−0.68

*—Shown as the normalized z-score of raw tightness values.

## Supplementary Materials

The following are available online at https://www.mdpi.com/1099-4300/22/10/1078/s1, Included with this pdf file.
