# Supplementary material for: Inferring Networks of Interdependent Labor Skills to Illuminate Urban Economic Structure
Source: Entropy (Basel). 2020 Sep 25;22(10):1078. doi: 10.3390/e22101078 (PMC7597157; doi:10.3390/e22101078)
Supplement: Supplementary file 1 [file entropy-22-01078-s001.pdf]

Supplementary Materials for

Inferring networks of interdependent labor skills to illuminate urban economic structure

Shade T. Shutters [1,*] and Keith Waters[2]

[1] Arizona State University; shade.shutters@asu.edu
[2] George Mason University; kwaters2@gmu.edu
* Correspondence: shade.shutters@asu.edu

This PDF file includes:

Section S1. Further network analysis/visualizations
Section S2. Mapping of O*NET occupation codes to BLS occupation codes
Section S3. List of MSAs and codes, NECTA exceptions, and their tightness and GDP values
Section S4. List of O*NET elements and IWAs and their communities as identified with the Louvain community detection algorithm
Section S5. Further statistical analysis
Supplemental references
Figure S1-1. Importance Network
Table S1-1. General Network Statistics
Table S1-2. Degree Centrality – Top Five Nodes
Table S1-3. Closeness Centrality – Top Five Nodes
Table S1-4. Betweenness Centrality – Top Five Nodes
Table S3-1. Mapping of NECTAs used for BLS labor data to MSAs used for BEA GDP data
Table S3-2. Mapping of old MSA still used by BLS to current MSAs used by BEA
Table S4-1. O*Net elements having an occupational value for level and their cluster assignment using the Louvain community detection algorithm
Table S4-2. Complete list of O*Net individual work activities (IWAs) and their cluster as determined by the Louvain community detection algorithm
Table S5-1. Regressions Explaining MSA GDP Per Capita 2018

S1. Further network analysis/visualizations

Importance Network

There are two different measure of the O*Net elements, levels and importance. Despite measuring different aspects of the elements, the resulting networks are qualitatively similar. Figure S1-1 displays the network created from the importance measure of the element. We applied the Louvain community detection algorithm (1) and, like the element network, found three communities. After removing edges less than zero, the overall network is polarized into two main groups, "sensory-physical" and "socio-cognitive" skills. As with the network created using the level measure instead of importance, the socio-cognitive lobe was found to have two sub-components illustrative of technical skills (green) and more general socio-cognitive skills (red). Sensory-physical elements are yellow.

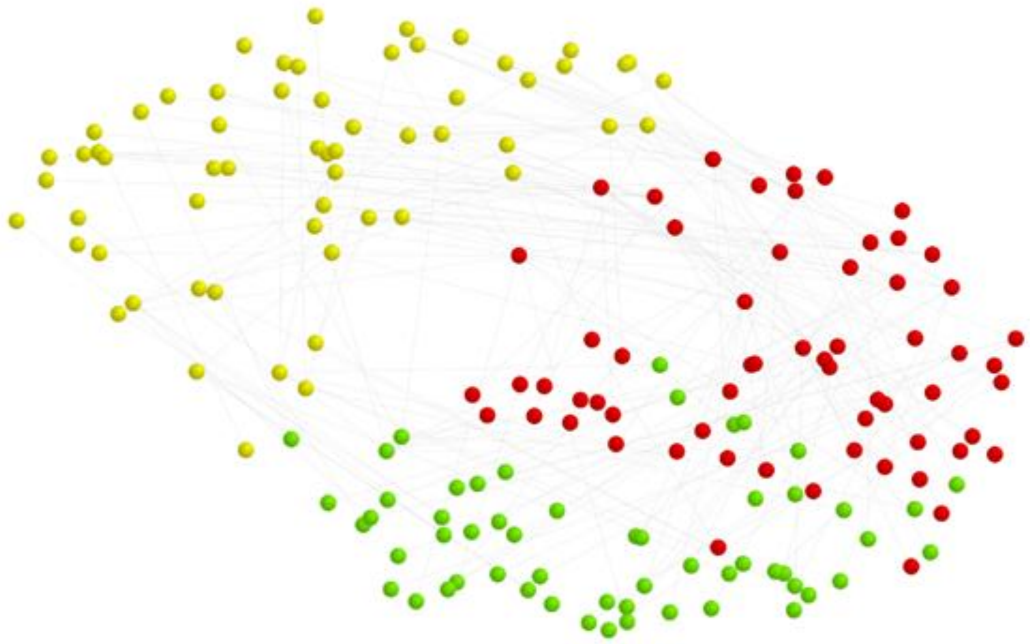

Figure S1-1. Importance Network. Elements are nodes with color indicating community: yellow = sensory-physical skills; green = technical socio-cognitive skills; and red = general socio-cognitive skills.

Network Statistics

Looking at two broad measures, density and average degree, further supports that the two element networks are similar to one another and different from the IWA network (Table S1-1). Consistent with reported figures, the networks analyzed exclude edges with weights below zero. Density is the number of edges present as a share of all possible edges and average degree

is the average of how many edges each node has. The two element networks have slightly over half of all possible edge present while the IWA network has slightly under half of all possible edges present. The average degree of the element networks, however, is substantially lower than the IWA network which has substantially more nodes than the element networks.

Table S1-1. General Network Statistics

Network	Density (No Loops Allowed)	Average Degree
Element: Level	0.518	82.894
Element: Importance	0.519	91.356
IWA	0.470	155.494
Note: Networks exclude edge weights < 0		

For each network, the nodes (elements or IWAs) with the highest degrees are reported in table S1-2. A nodes degree is simply the number of other nodes it is connected to. While the two element networks are visually similar to one another, the top five nodes differ with only oral comprehension appearing in both.

Table S1-2. Degree Centrality – Top Five Nodes*

Rank	Element: Level		Element: Importance		IWA	
	Element	Degree	Element	Degree	IWA	Degree
1	Speech Clarity (1.A.4.b.5)	101	Oral Comprehension (1.A.1.a.1)	115	Take physical measurements of patients or clients. (4.A.1.b.3.I05)	217
2	Time Management (2.B.5.a)	101	Oral Expression (1.A.1.a.3)	114	Train animals. (4.A.3.a.2.I10)	216
3	Resolving Conflicts and Negotiating with Others (4.A.4.a.7)	101	Memorization (1.A.1.d.1)	114	Assist individuals with paperwork. (4.A.4.a.5.I08)	215
4	Performing Administrative Activities (4.A.4.c.1)	101	Speech Clarity (1.A.4.b.5)	114	Consult legal materials or public records. (4.A.1.a.1.I05)	212
5	Oral Comprehension (1.A.1.a.1)	100	Integrity (1.C.5.c)	114	Discuss legal matters with clients, disputants, or legal professionals or staff. (4.A.4.a.2.I12)	209
*Note that additional nodes share the degree score and only five are shown for each.						

The closeness centrality of a node is a measure of distance to all other nodes. The 5 nodes with the highest closeness centrality are reported in table S1-3. Closeness centrality for any given node is the number of nodes divided by the sum of all the shortest path lengths from that node to all other nodes. The measure ranges from zero to one with one representing a direct link to all other nodes. Closeness centrality has been identified as a measure of the ease at which a node can obtain information (2). In contrast to degree, the two element networks have three common nodes among the highest in the network: speech clarity, oral comprehension, and oral expression. Nodes in the socio-cognitive: general group account for nine of the top ten nodes in terms of element (level) closeness centrality. In stark contrast, nodes in the sensory-physical community account entirely for the nodes with the lowest closeness centrality for levels.

Table S1-3. Closeness Centrality – Top Five Nodes*

Rank	Element: Level		Element: Importance		IWA	
	Element	Closeness Centrality	Element	Closeness Centrality	IWA	Closeness Centrality
1	Resolving Conflicts and Negotiating with Others (4.A.4.a.7)	0.730594	Oral Comprehension (1.A.1.a.1)	0.74262	Take physical measurements of patients or clients. (4.A.1.b.3.I05)	0.74382
2	Time Management (2.B.5.a)	0.730594	Oral Expression (1.A.1.a.3)	0.73950	Train animals. (4.A.3.a.2.I10)	0.74215
3	Speech Clarity (1.A.4.b.5)	0.727273	Memorization (1.A.1.d.1)	0.73950	Assist individuals with paperwork. (4.A.4.a.5.I08)	0.74049
4	Oral Comprehension (1.A.1.a.1)	0.727273	Speech Clarity (1.A.4.b.5)	0.73950	Consult legal materials or public records. (4.A.1.a.1.I05)	0.73556
5	Oral Expression (1.A.1.a.3)	0.727273	Active Listening (2.A.1.b)	0.73950	Discuss legal matters with clients, disputants, or legal professionals or staff. (4.A.4.a.2.I12)	0.73068

*Note that additional nodes share the closeness centrality score and only five are shown for each.

Finally, the highest betweenness centrality scores are reported in table S1-4. Betweenness centrality measures the share of shortest paths that include the node. The highest betweenness measures are notably higher in the element networks, reflecting the polarized nature of these networks. The nodes with the highest betweenness are located at the narrow points in between the two polarized portions of the network. These are skills that may be able to bridge the gap between the polarized regions of the networks and allow workers with these skills to easily transition between the two substantially different portions of the network.

Table S1-4. Betweenness Centrality – Top Five Nodes

Rank	Element: Level		Element: Importance		IWA	
	Element	Betweenness Centrality	Element	Betweenness Centrality	IWA	Betweenness Centrality
1	Installation (2.B.3.d)	0.027037	Estimating the Quantifiable Characteristics of Products, Events, or Information (4.A.1.b.3)	0.02209	Assist individuals with paperwork. (4.A.4.a.5.I08)	0.005192
2	Physics (2.C.4.b)	0.02436	Physics (2.C.4.b)	0.02131	Train animals. (4.A.3.a.2.I10)	0.005010
3	Coordination (2.B.1.b)	0.022365	Visualization (1.A.1.f.2)	0.01916	Take physical measurements of patients or clients. (4.A.1.b.3.I05)	0.004607
4	Drafting, Laying Out, and Specifying Technical Devices, Parts, and Equipment (4.A.3.b.2)	0.021724	Selective Attention (1.A.1.g.1)	0.01702	Engrave objects. (4.A.3.a.2.I25)	0.004560
5	Engineering and Technology (2.C.3.b)	0.02096	Drafting, Laying Out, and Specifying Technical Devices, Parts, and Equipment (4.A.3.b.2)	0.01620	Evaluate the quality or accuracy of data. (4.A.2.a.2.I01)	0.004469

S2. Mapping O*NET occupation codes to BLS occupation codes

In our analysis we paired O*NET version 24.2 (3) with the Bureau of Labor Statistics (BLS) 2018 occupational employment statistics (4). These two entities use slightly different occupation codes. In particular, the BLS uses the federal standard 6-digit code, while O*NET adds an extra 2-digits so that occupations may be further divided. Here we describe in detail our procedure for linking the two data sets.

With two exceptions, every O*NET occupation code can be mapped to one BLS occupation code. In most cases, one O*NET code maps to one and only one BLS code, as in the following example:

O*NET code	O*Net name	BLS code	BLS name
11-3011.00	Administrative Services Managers	11-3011	Administrative Services Managers

In some cases, multiple O*NET occupations map to a single BLS occupation. In those cases, we take the average of an element's values across the multiple O*NET codes and assign it to the single corresponding BLS code as in the following example:

O*NET code	O*Net name	BLS code	BLS name
11-3051.00<br>11-3051.01<br>11-3051.02<br>11-3051.03<br>11-3051.04	Industrial Production Managers<br>Quality Control Systems Managers<br>Geothermal Production Managers<br>Biofuels Production Managers<br>Biomass Power Plant Managers	11-3011	Industrial Production Managers

This leaves the two exceptions requiring further processing. O*NET version 24.2 still uses one occupation code that the was retired by the BLS after its 2016 data release: 25-3099 - Miscellaneous Teaching Occupations. In 2017 the BLS replaced this occupation code with two new codes, 25-3097 - Teachers and Instructors, All Other, Except Substitute Teachers, and 25-3098 - Substitute Teachers. O*Net 24.2 continues to use the older code. Therefore, first map O*Net values to the old BLS code 25-3099, and then apply those element averages to the two BLS occupations that replaced 25-3099, namely 25-3097 and 25-3098.

The full mapping is included in the accompanying file: bls_onet_crosswalk_2018.csv

S3. Mapping Metropolitan Statistical Areas

Despite using the term metropolitan statistical area (MSA) to describe its geographical areas of aggregation, the Bureau of Labor (BLS) statistics actually uses an alternative geospatial unit in the six New England states. These units are known at New England City and Town Areas or NECTAs (5). This presents problems of matching labor data to almost every other federal data set, including GDP data we take from the U.S. Bureau of Labor Statistics (BEA). Thus, we created a mapping of areas used by the BLS to corresponding areas used by the BEA. These units have similar names but do not cover the same geographical areas. However, as we are using rates (e.g. per capita GDP) and not gross numbers, we accept these as generally applicable to their NECTA counterparts. Table S3-2 presents the metropolitan areas used by the BLS (NECTAs) to aggregate labor data and how we mapped them to metropolitan areas used by the BEA (MSAs).

Table S3-1. Mapping of NECTAs used for BLS labor data to MSAs used for BEA GDP data

NECTA (BLS)		MSA (BEA)	
**Code**	**Name**	**Code**	**Name**
70750	Bangor, ME	12620	Bangor, ME
70900	Barnstable Town, MA	12700	Barnstable Town, MA
71650	Boston-Cambridge-Nashua, MA-NH	14460	Boston-Cambridge-Newton, MA-NH
71950	Bridgeport-Stamford-Norwalk, CT	14860	Bridgeport-Stamford-Norwalk, CT
72400	Burlington-South Burlington, VT	15540	Burlington-South Burlington, VT
72850	Danbury, CT		*
73050	Dover-Durham, NH-ME		*
73450	Hartford-West Hartford-East Hartford, CT	25540	Hartford-East Hartford-Middletown, CT
74500	Leominster-Gardner, MA		*
74650	Lewiston-Auburn, ME	30340	Lewiston-Auburn, ME
74950	Manchester, NH	31700	Manchester-Nashua, NH
75550	New Bedford, MA		*
75700	New Haven, CT	35300	New Haven-Milford, CT
76450	Norwich-New London-Westerly, CT-RI	35980	Norwich-New London, CT
76600	Pittsfield, MA	38340	Pittsfield, MA
76750	Portland-South Portland, ME	38860	Portland-South Portland, ME
76900	Portsmouth, NH-ME		*
77200	Providence-Warwick, RI-MA	39300	Providence-Warwick, RI-MA
78100	Springfield, MA-CT	44140	Springfield, MA
78700	Waterbury, CT		*
79600	Worcester, MA-CT	49340	Worcester, MA-CT

* NECTA has no corresponding MSA and thus no GDP data is reported

In addition, the BLS continues to use two old MSA codes that have been renamed by Office of Management and Budget, and which are used by the BEA. Our mapping of these old to current codes is shown in Table S3-2.

Table S3-2. Mapping of old MSA still used by BLS to current MSAs used by BEA

Old MSA (BLS)		Current MSAs (BEA)	
**Code**	**Name**	**Code**	**Name**
19380	Dayton, OH	19430	Dayton-Kettering, OH
39140	Prescott, AZ	39150	Prescott Valley-Prescott, AZ

Finally, the BEA does not report GDP values for MSAs in Puerto Rico, thus those MSAs have labor data but no corresponding GDP data and are dropped for purposes of correlations between tightness and GDP.

S4. List of O*NET elements and individual work activities (IWAs) used

The O*Net dataset decomposes occupations into a set of attributes called elements, which are assigned values for dimensions such as importance and level. The full list of these elements is shown in Table S4-1. O*Net additional decomposes occupations into a set of individual work activities (IWAs), which are either present or absent for each occupation. The complete list of IWAs is presented in Table S4-2. In both tables, each element's assigned cluster, as determined by the Louvain community detection algorithm, is show.

Table S4-1. O*Net elements having an occupational value for level and their cluster assignment using the Louvain community detection algorithm

Cluster	Element id	Element name
1	1.A.1.a.1	Oral Comprehension
1	1.A.1.a.3	Oral Expression
1	1.A.1.a.4	Written Expression
1	1.A.1.b.3	Problem Sensitivity
1	1.A.1.b.5	Inductive Reasoning
1	1.A.1.b.6	Information Ordering
1	1.A.1.b.7	Category Flexibility
1	1.A.1.d.1	Memorization
1	1.A.1.e.1	Speed of Closure
1	1.A.4.a.1	Near Vision
1	1.A.4.b.4	Speech Recognition
1	1.A.4.b.5	Speech Clarity
1	2.A.1.b	Active Listening
1	2.A.1.c	Writing
1	2.A.1.d	Speaking
1	2.A.2.a	Critical Thinking
1	2.A.2.b	Active Learning
1	2.A.2.c	Learning Strategies
1	2.A.2.d	Monitoring
1	2.B.1.a	Social Perceptiveness
1	2.B.1.b	Coordination
1	2.B.1.c	Persuasion
1	2.B.1.d	Negotiation
1	2.B.1.e	Instructing
1	2.B.1.f	Service Orientation
1	2.B.5.a	Time Management
1	2.B.5.d	Management of Personnel Resources
1	2.C.1.b	Clerical
1	2.C.1.d	Sales and Marketing
1	2.C.1.e	Customer and Personal Service
1	2.C.4.d	Biology
1	2.C.4.e	Psychology
1	2.C.4.f	Sociology and Anthropology

1	2.C.5.a	Medicine and Dentistry
1	2.C.5.b	Therapy and Counseling
1	2.C.6	Education and Training
1	2.C.7.a	English Language
1	2.C.7.b	Foreign Language
1	2.C.7.c	Fine Arts
1	2.C.7.d	History and Archeology
1	2.C.7.e	Philosophy and Theology
1	4.A.1.b.1	Identifying Objects, Actions, and Events
1	4.A.2.b.6	Organizing, Planning, and Prioritizing Work
1	4.A.4.a.4	Establishing and Maintaining Interpersonal Relationships
1	4.A.4.a.5	Assisting and Caring for Others
1	4.A.4.a.6	Selling or Influencing Others
1	4.A.4.a.7	Resolving Conflicts and Negotiating with Others
1	4.A.4.a.8	Performing for or Working Directly with the Public
1	4.A.4.b.3	Training and Teaching Others
1	4.A.4.b.5	Coaching and Developing Others
1	4.A.4.c.1	Performing Administrative Activities
2	1.A.1.a.2	Written Comprehension
2	1.A.1.b.1	Fluency of Ideas
2	1.A.1.b.2	Originality
2	1.A.1.b.4	Deductive Reasoning
2	1.A.1.c.1	Mathematical Reasoning
2	1.A.1.c.2	Number Facility
2	1.A.1.e.2	Flexibility of Closure
2	2.A.1.a	Reading Comprehension
2	2.A.1.e	Mathematics
2	2.A.1.f	Science
2	2.B.2.i	Complex Problem Solving
2	2.B.3.a	Operations Analysis
2	2.B.3.b	Technology Design
2	2.B.3.e	Programming
2	2.B.4.e	Judgment and Decision Making
2	2.B.4.g	Systems Analysis
2	2.B.4.h	Systems Evaluation
2	2.B.5.b	Management of Financial Resources
2	2.B.5.c	Management of Material Resources
2	2.C.1.a	Administration and Management
2	2.C.1.c	Economics and Accounting
2	2.C.1.f	Personnel and Human Resources
2	2.C.3.a	Computers and Electronics
2	2.C.3.b	Engineering and Technology
2	2.C.3.c	Design
2	2.C.4.a	Mathematics
2	2.C.4.g	Geography
2	2.C.8.b	Law and Government
2	2.C.9.a	Telecommunications
2	2.C.9.b	Communications and Media
2	4.A.1.a.1	Getting Information

2	4.A.1.b.3	Estimating the Quantifiable Characteristics of Products, Events, or Information
2	4.A.2.a.1	Judging the Qualities of Things, Services, or People
2	4.A.2.a.2	Processing Information
2	4.A.2.a.3	Evaluating Information to Determine Compliance with Standards
2	4.A.2.a.4	Analyzing Data or Information
2	4.A.2.b.1	Making Decisions and Solving Problems
2	4.A.2.b.2	Thinking Creatively
2	4.A.2.b.3	Updating and Using Relevant Knowledge
2	4.A.2.b.4	Developing Objectives and Strategies
2	4.A.2.b.5	Scheduling Work and Activities
2	4.A.3.b.1	Interacting With Computers
2	4.A.3.b.6	Documenting/Recording Information
2	4.A.4.a.1	Interpreting the Meaning of Information for Others
2	4.A.4.a.2	Communicating with Supervisors, Peers, or Subordinates
2	4.A.4.a.3	Communicating with Persons Outside Organization
2	4.A.4.b.1	Coordinating the Work and Activities of Others
2	4.A.4.b.2	Developing and Building Teams
2	4.A.4.b.4	Guiding, Directing, and Motivating Subordinates
2	4.A.4.b.6	Provide Consultation and Advice to Others
2	4.A.4.c.2	Staffing Organizational Units
2	4.A.4.c.3	Monitoring and Controlling Resources
3	1.A.1.e.3	Perceptual Speed
3	1.A.1.f.1	Spatial Orientation
3	1.A.1.f.2	Visualization
3	1.A.1.g.1	Selective Attention
3	1.A.1.g.2	Time Sharing
3	1.A.2.a.1	Arm-Hand Steadiness
3	1.A.2.a.2	Manual Dexterity
3	1.A.2.a.3	Finger Dexterity
3	1.A.2.b.1	Control Precision
3	1.A.2.b.2	Multilimb Coordination
3	1.A.2.b.3	Response Orientation
3	1.A.2.b.4	Rate Control
3	1.A.2.c.1	Reaction Time
3	1.A.2.c.2	Wrist-Finger Speed
3	1.A.2.c.3	Speed of Limb Movement
3	1.A.3.a.1	Static Strength
3	1.A.3.a.2	Explosive Strength
3	1.A.3.a.3	Dynamic Strength
3	1.A.3.a.4	Trunk Strength
3	1.A.3.b.1	Stamina
3	1.A.3.c.1	Extent Flexibility
3	1.A.3.c.2	Dynamic Flexibility
3	1.A.3.c.3	Gross Body Coordination
3	1.A.3.c.4	Gross Body Equilibrium
3	1.A.4.a.2	Far Vision
3	1.A.4.a.3	Visual Color Discrimination
3	1.A.4.a.4	Night Vision
3	1.A.4.a.5	Peripheral Vision

3	1.A.4.a.6	Depth Perception
3	1.A.4.a.7	Glare Sensitivity
3	1.A.4.b.1	Hearing Sensitivity
3	1.A.4.b.2	Auditory Attention
3	1.A.4.b.3	Sound Localization
3	2.B.3.c	Equipment Selection
3	2.B.3.d	Installation
3	2.B.3.g	Operation Monitoring
3	2.B.3.h	Operation and Control
3	2.B.3.j	Equipment Maintenance
3	2.B.3.k	Troubleshooting
3	2.B.3.l	Repairing
3	2.B.3.m	Quality Control Analysis
3	2.C.10	Transportation
3	2.C.2.a	Production and Processing
3	2.C.2.b	Food Production
3	2.C.3.d	Building and Construction
3	2.C.3.e	Mechanical
3	2.C.4.b	Physics
3	2.C.4.c	Chemistry
3	2.C.8.a	Public Safety and Security
3	4.A.1.a.2	Monitor Processes, Materials, or Surroundings
3	4.A.1.b.2	Inspecting Equipment, Structures, or Material
3	4.A.3.a.1	Performing General Physical Activities
3	4.A.3.a.2	Handling and Moving Objects
3	4.A.3.a.3	Controlling Machines and Processes
3	4.A.3.a.4	Operating Vehicles, Mechanized Devices, or Equipment
3	4.A.3.b.2	Drafting, Laying Out, and Specifying Technical Devices, Parts, and Equipment
3	4.A.3.b.4	Repairing and Maintaining Mechanical Equipment
3	4.A.3.b.5	Repairing and Maintaining Electronic Equipment

Table S4-2. Complete list of O*Net individual work activities (IWAs) and their cluster as determined by the Louvain community detection algorithm.

Cluster	IWA ID	IWA name
1	4.A.1.a.1.I02	Read documents or materials to inform work processes.
1	4.A.1.a.2.I01	Monitor equipment operation.
1	4.A.1.a.2.I02	Monitor operations to ensure adequate performance.
1	4.A.1.b.1.I01	Mark materials or objects for identification.
1	4.A.1.b.2.I03	Test characteristics of materials or products.
1	4.A.1.b.2.I04	Inspect vehicles.
1	4.A.1.b.2.I05	Inspect facilities or equipment.
1	4.A.1.b.2.I06	Inspect completed work or finished products.
1	4.A.1.b.2.I07	Inspect commercial, industrial, or production systems or equipment.
1	4.A.1.b.2.I10	Inspect characteristics or conditions of materials or products.
1	4.A.1.b.2.I11	Test sites or materials for environmental hazards.

1	4.A.1.b.2.I12	Test performance of equipment or systems.
1	4.A.1.b.3.I01	Measure physical characteristics of materials, products, or equipment.
1	4.A.1.b.3.I04	Assess characteristics of land or property.
1	4.A.2.a.1.I05	Evaluate production inputs or outputs.
1	4.A.2.a.2.I02	Sort materials or products.
1	4.A.2.b.1.I02	Diagnose system or equipment problems.
1	4.A.2.b.1.I04	Determine operational methods or procedures.
1	4.A.2.b.1.I10	Select materials or equipment for operations or projects.
1	4.A.2.b.2.I13	Design materials or devices.
1	4.A.2.b.2.I14	Design industrial systems or equipment.
1	4.A.2.b.6.I02	Plan work activities.
1	4.A.3.a.1.I01	Prepare mixtures or solutions.
1	4.A.3.a.1.I03	Clean tools, equipment, facilities, or work areas.
1	4.A.3.a.1.I04	Set up protective structures or coverings near work areas.
1	4.A.3.a.1.I05	Dispose of waste or debris.
1	4.A.3.a.1.I06	Load products, materials, or equipment for transportation or further processing.
1	4.A.3.a.1.I07	Perform general construction or extraction activities.
1	4.A.3.a.1.I08	Clean workpieces, finished products, or other objects.
1	4.A.3.a.1.I10	Move materials, equipment, or supplies.
1	4.A.3.a.2.I01	Build structures.
1	4.A.3.a.2.I02	Collect samples of products or materials.
1	4.A.3.a.2.I04	Assemble equipment or components.
1	4.A.3.a.2.I05	Sew garments or materials.
1	4.A.3.a.2.I06	Position workpieces or materials on equipment.
1	4.A.3.a.2.I07	Shape materials to create products.
1	4.A.3.a.2.I09	Adjust equipment to ensure adequate performance.
1	4.A.3.a.2.I12	Cut materials.
1	4.A.3.a.2.I13	Prepare industrial materials for processing or use.
1	4.A.3.a.2.I15	Smooth surfaces of objects or equipment.
1	4.A.3.a.2.I16	Install energy or heating equipment.
1	4.A.3.a.2.I19	Disassemble equipment.
1	4.A.3.a.2.I20	Install commercial or production equipment.
1	4.A.3.a.2.I22	Position tools or equipment.
1	4.A.3.a.2.I23	Tend watercraft.
1	4.A.3.a.2.I25	Engrave objects.
1	4.A.3.a.2.I26	Embalm corpses.
1	4.A.3.a.2.I27	Fabricate devices or components.
1	4.A.3.a.2.I28	Create decorative objects or parts of objects.
1	4.A.3.a.2.I29	Process animal carcasses.
1	4.A.3.a.2.I30	Drill holes in earth or materials.
1	4.A.3.a.2.I33	Apply protective solutions or coatings.
1	4.A.3.a.2.I34	Connect components or supply lines to equipment or tools.
1	4.A.3.a.2.I35	Join parts using soldering, welding, or brazing techniques.
1	4.A.3.a.2.I36	Assemble products or work aids.
1	4.A.3.a.2.I39	Apply materials to fill gaps or imperfections.
1	4.A.3.a.2.I40	Remove workpieces from production equipment.

1	4.A.3.a.2.I41	Position materials or components for assembly.
1	4.A.3.a.2.I42	Package objects.
1	4.A.3.a.2.I43	Install plumbing or piping equipment or systems.
1	4.A.3.a.2.I45	Apply decorative finishes.
1	4.A.3.a.2.I46	Set up equipment.
1	4.A.3.a.3.I02	Operate pumping systems or equipment.
1	4.A.3.a.3.I03	Operate construction or excavation equipment.
1	4.A.3.a.3.I06	Operate industrial processing or production equipment.
1	4.A.3.a.3.I07	Operate energy production or distribution equipment.
1	4.A.3.a.3.I08	Operate lifting or moving equipment.
1	4.A.3.a.3.I12	Operate cutting or grinding equipment.
1	4.A.3.a.4.I01	Operate transportation equipment or vehicles.
1	4.A.3.b.4.I01	Maintain facilities or equipment.
1	4.A.3.b.4.I02	Maintain tools or equipment.
1	4.A.3.b.4.I03	Repair electrical or electronic equipment.
1	4.A.3.b.4.I05	Repair workpieces or products.
1	4.A.3.b.4.I06	Repair tools or equipment.
1	4.A.3.b.4.I07	Repair vehicle components.
1	4.A.3.b.4.I08	Maintain vehicles in working condition.
1	4.A.3.b.6.I08	Maintain operational records.
1	4.A.4.a.2.I03	Communicate with others about operational plans or activities.
1	4.A.4.a.2.I08	Notify others of emergencies or problems.
1	4.A.4.a.2.I10	Signal others to coordinate work activities.
1	4.A.4.a.8.I02	Conduct amusement or gaming activities.
2	4.A.1.a.1.I01	Study details of artistic productions.
2	4.A.1.a.1.I03	Investigate criminal or legal matters.
2	4.A.1.a.1.I05	Consult legal materials or public records.
2	4.A.1.a.1.I11	Gather information for news stories.
2	4.A.1.a.1.I14	Collect data about consumer needs or opinions.
2	4.A.1.a.1.I15	Investigate individuals' background, behavior, or activities.
2	4.A.1.a.1.I23	Interview people to obtain information.
2	4.A.1.a.2.I04	Monitor traffic conditions.
2	4.A.1.a.2.I08	Monitor external affairs, trends, or events.
2	4.A.1.b.3.I03	Calculate financial data.
2	4.A.1.b.3.I05	Take physical measurements of patients or clients.
2	4.A.2.a.1.I02	Evaluate programs, practices, or processes.
2	4.A.2.a.1.I04	Evaluate personnel capabilities or performance.
2	4.A.2.a.1.I08	Evaluate scholarly work.
2	4.A.2.a.2.I01	Evaluate the quality or accuracy of data.
2	4.A.2.a.2.I03	Compile records, documentation, or other data.
2	4.A.2.a.3.I03	Examine financial activities, operations, or systems.
2	4.A.2.a.4.I08	Research laws, precedents, or other legal data.
2	4.A.2.b.1.I05	Make legal decisions.
2	4.A.2.b.1.I07	Edit written materials or documents.
2	4.A.2.b.1.I11	Alter audio or video recordings.
2	4.A.2.b.2.I22	Develop news, entertainment, or artistic content.

2	4.A.2.b.2.I25	Create artistic designs or performances.
2	4.A.2.b.4.I02	Draft legislation or regulations.
2	4.A.2.b.6.I01	Plan events or programs.
2	4.A.3.a.1.I14	Climb equipment or structures.
2	4.A.3.a.2.I21	Apply hygienic or cosmetic agents to skin or hair.
2	4.A.3.a.2.I31	Groom or style hair.
2	4.A.3.b.1.I06	Process digital or online data.
2	4.A.3.b.6.I02	Record information about legal matters.
2	4.A.3.b.6.I12	Prepare informational or instructional materials.
2	4.A.3.b.6.I16	Write material for artistic or commercial purposes.
2	4.A.4.a.1.I02	Explain regulations, policies, or procedures.
2	4.A.4.a.1.I03	Interpret language, cultural, or religious information for others.
2	4.A.4.a.1.I04	Explain financial information.
2	4.A.4.a.2.I12	Discuss legal matters with clients, disputants, or legal professionals or staff.
2	4.A.4.a.3.I01	Present information in legal proceedings.
2	4.A.4.a.5.I05	Intervene in crisis situations or emergencies.
2	4.A.4.a.5.I08	Assist individuals with paperwork.
2	4.A.4.a.6.I03	Promote products, services, or programs.
2	4.A.4.a.7.I01	Mediate disputes.
2	4.A.4.a.8.I01	Present arts or entertainment performances.
2	4.A.4.b.4.I03	Coordinate artistic or entertainment activities.
2	4.A.4.b.4.I12	Direct organizational operations, activities, or procedures.
2	4.A.4.b.4.I15	Direct security or safety activities or operations.
2	4.A.4.b.5.I01	Coach others.
2	4.A.4.c.2.I02	Perform human resources activities.
3	4.A.1.a.1.I04	Gather information from physical or electronic sources.
3	4.A.1.a.1.I12	Collect information about patients or clients.
3	4.A.1.a.1.I18	Research historical or social issues.
3	4.A.1.a.1.I19	Research healthcare issues.
3	4.A.1.a.1.I21	Investigate incidents or accidents.
3	4.A.1.a.2.I03	Monitor financial data or activities.
3	4.A.1.a.2.I05	Monitor health conditions of humans or animals.
3	4.A.1.a.2.I06	Monitor individual behavior or performance.
3	4.A.1.a.2.I07	Monitor safety or security of work areas, facilities, or properties.
3	4.A.1.b.2.I01	Administer diagnostic tests to assess patient health.
3	4.A.1.b.2.I09	Examine people or animals to assess health conditions or physical characteristics.
3	4.A.2.a.1.I01	Assess living, work, or social needs or status of individuals or communities.
3	4.A.2.a.1.I03	Assess student capabilities, needs, or performance.
3	4.A.2.a.1.I06	Evaluate patient or client condition or treatment options.
3	4.A.2.a.2.I04	Reconcile financial data.
3	4.A.2.a.3.I01	Examine materials or documentation for accuracy or compliance.
3	4.A.2.a.3.I04	Follow standard healthcare safety procedures to protect patient and staff members.
3	4.A.2.a.4.I06	Analyze health or medical data.
3	4.A.2.b.1.I01	Determine values or prices of goods or services.
3	4.A.2.b.1.I06	Diagnose health conditions or disorders.
3	4.A.2.b.2.I01	Develop patient or client care or treatment plans.

3	4.A.2.b.2.I04	Develop recipes or menus.
3	4.A.2.b.2.I05	Develop safety standards, policies, or procedures.
3	4.A.2.b.2.I10	Develop health assessment methods or programs.
3	4.A.2.b.2.I15	Develop educational programs, plans, or procedures.
3	4.A.2.b.2.I17	Develop public or community health programs.
3	4.A.2.b.2.I27	Develop organizational or program goals or objectives.
3	4.A.2.b.5.I01	Prepare schedules for services or facilities.
3	4.A.2.b.5.I02	Schedule operational activities.
3	4.A.2.b.5.I03	Schedule appointments.
3	4.A.3.a.1.I02	Protect people or property from threats such as fires or flooding.
3	4.A.3.a.1.I09	Clean medical equipment or facilities.
3	4.A.3.a.1.I11	Maintain safety or security.
3	4.A.3.a.1.I12	Escort others.
3	4.A.3.a.1.I13	Transport patients or clients.
3	4.A.3.a.1.I15	Provide food or beverage services.
3	4.A.3.a.1.I16	Perform athletic activities for fitness, competition, or artistic purposes.
3	4.A.3.a.1.I17	Perform agricultural activities.
3	4.A.3.a.2.I03	Prepare foods or beverages.
3	4.A.3.a.2.I08	Arrange displays or decorations.
3	4.A.3.a.2.I10	Train animals.
3	4.A.3.a.2.I11	Prepare medical equipment or work areas for use.
3	4.A.3.a.2.I14	Cut trees or other vegetation.
3	4.A.3.a.2.I17	Fabricate medical devices.
3	4.A.3.a.2.I18	Direct vehicle traffic.
3	4.A.3.a.2.I24	Adjust medical equipment to ensure adequate performance.
3	4.A.3.a.2.I32	Set up classrooms, facilities, educational materials, or equipment.
3	4.A.3.a.2.I37	Stock supplies or products.
3	4.A.3.a.2.I38	Collect environmental or biological samples.
3	4.A.3.a.2.I44	Hunt animals.
3	4.A.3.a.2.I47	Prepare specimens or materials for testing.
3	4.A.3.a.3.I01	Operate office equipment.
3	4.A.3.a.3.I04	Operate medical equipment.
3	4.A.3.a.3.I05	Operate audiovisual or related equipment.
3	4.A.3.a.3.I10	Operate communications equipment or systems.
3	4.A.3.a.3.I11	Operate agricultural or forestry equipment.
3	4.A.3.b.1.I05	Operate computer systems or computerized equipment.
3	4.A.3.b.4.I09	Maintain medical equipment or instruments.
3	4.A.3.b.6.I04	Record information about environmental conditions.
3	4.A.3.b.6.I05	Record images with photographic or audiovisual equipment.
3	4.A.3.b.6.I06	Prepare health or medical documents.
3	4.A.3.b.6.I10	Maintain sales or financial records.
3	4.A.3.b.6.I11	Maintain health or medical records.
3	4.A.3.b.6.I13	Prepare documentation for contracts, applications, or permits.
3	4.A.4.a.1.I01	Explain technical details of products or services.
3	4.A.4.a.1.I05	Explain medical information to patients or family members.
3	4.A.4.a.2.I02	Assist scientists, scholars, or technical specialists with projects or research.

3	4.A.4.a.2.I05	Confer with healthcare or other professionals about patient care.
3	4.A.4.a.2.I07	Collaborate in the development of educational programs.
3	4.A.4.a.3.I02	Provide information or assistance to the public.
3	4.A.4.a.3.I03	Provide information to guests, clients, or customers.
3	4.A.4.a.5.I01	Assist others to access additional services or resources.
3	4.A.4.a.5.I02	Administer therapeutic treatments.
3	4.A.4.a.5.I03	Administer basic health care or medical treatments.
3	4.A.4.a.5.I04	Care for plants or animals.
3	4.A.4.a.5.I06	Fit assistive devices to patients or clients.
3	4.A.4.a.5.I07	Administer emergency medical treatment.
3	4.A.4.a.5.I09	Assist individuals with special needs.
3	4.A.4.a.5.I10	Provide general assistance to others, such as customers, patrons, or motorists.
3	4.A.4.a.5.I11	Assist healthcare practitioners during medical procedures.
3	4.A.4.a.5.I12	Treat injuries, illnesses, or diseases.
3	4.A.4.a.6.I01	Advocate for individual or community needs.
3	4.A.4.a.6.I02	Sell products or services.
3	4.A.4.a.8.I03	Respond to customer problems or inquiries.
3	4.A.4.b.3.I01	Teach life skills.
3	4.A.4.b.3.I02	Teach academic or vocational subjects.
3	4.A.4.b.3.I03	Teach safety procedures or standards to others.
3	4.A.4.b.3.I04	Train others on operational or work procedures.
3	4.A.4.b.3.I06	Train others on health or medical topics.
3	4.A.4.b.4.I02	Serve on organizational committees.
3	4.A.4.b.4.I04	Manage agricultural or forestry operations.
3	4.A.4.b.4.I05	Supervise activities in correctional facilities.
3	4.A.4.b.4.I11	Coordinate group, community, or public activities.
3	4.A.4.b.4.I13	Assign work to others.
3	4.A.4.b.4.I16	Direct legal activities.
3	4.A.4.b.5.I02	Provide support or encouragement to others.
3	4.A.4.b.6.I01	Advise patients or clients on medical issues.
3	4.A.4.b.6.I02	Advise others on products or services.
3	4.A.4.b.6.I06	Advise others on healthcare or wellness issues.
3	4.A.4.b.6.I07	Advise others on educational or vocational matters.
3	4.A.4.b.6.I09	Advise others on workplace health or safety issues.
3	4.A.4.b.6.I10	Counsel others about personal matters.
3	4.A.4.c.1.I01	Perform administrative or clerical activities.
3	4.A.4.c.1.I02	Perform court-related or other legal administrative activities.
3	4.A.4.c.1.I03	Execute financial transactions.
3	4.A.4.c.1.I04	Issue documentation.
3	4.A.4.c.1.I05	Process shipments or mail.
3	4.A.4.c.1.I06	Process forensic or legal evidence.
3	4.A.4.c.3.I01	Replenish inventories of materials, equipment, or products.
3	4.A.4.c.3.I02	Order medical tests or procedures.
3	4.A.4.c.3.I03	Distribute materials, supplies, or resources.
3	4.A.4.c.3.I04	Collect fares or payments.
3	4.A.4.c.3.I05	Purchase goods or services.

3	4.A.4.c.3.I06	Prescribe medical treatments or devices.
3	4.A.4.c.3.I07	Monitor resources or inventories.
4	4.A.1.a.1.I06	Gather data about operational or development activities.
4	4.A.1.a.1.I07	Obtain information about goods or services.
4	4.A.1.a.1.I08	Research issues related to earth sciences.
4	4.A.1.a.1.I09	Research organizational behavior, processes, or performance.
4	4.A.1.a.1.I10	Investigate the environmental impact of industrial or development activities.
4	4.A.1.a.1.I13	Research agricultural processes or practices.
4	4.A.1.a.1.I16	Research biological or ecological phenomena.
4	4.A.1.a.1.I17	Obtain formal documentation or authorization.
4	4.A.1.a.1.I20	Research technology designs or applications.
4	4.A.1.a.1.I22	Investigate organizational or operational problems.
4	4.A.1.a.2.I09	Monitor environmental conditions.
4	4.A.1.a.2.I10	Monitor operation of computer or information technologies.
4	4.A.1.a.2.I11	Monitor operations to ensure compliance with regulations or standards.
4	4.A.1.b.1.I02	Identify business or organizational opportunities.
4	4.A.1.b.2.I02	Evaluate green technologies or processes.
4	4.A.1.b.2.I08	Test performance of computer or information systems.
4	4.A.1.b.3.I02	Estimate project development or operational costs.
4	4.A.2.a.1.I07	Evaluate the characteristics, usefulness, or performance of products or technologies.
4	4.A.2.a.1.I09	Evaluate condition of financial assets, property, or other resources.
4	4.A.2.a.1.I10	Evaluate project feasibility.
4	4.A.2.a.2.I05	Verify personal information.
4	4.A.2.a.3.I02	Assess compliance with environmental standards or regulations.
4	4.A.2.a.4.I01	Analyze environmental or geospatial data.
4	4.A.2.a.4.I02	Analyze market or industry conditions.
4	4.A.2.a.4.I03	Analyze business or financial risks.
4	4.A.2.a.4.I04	Analyze scientific or applied data using mathematical principles.
4	4.A.2.a.4.I05	Evaluate designs, specifications, or other technical data.
4	4.A.2.a.4.I07	Analyze data to improve operations.
4	4.A.2.a.4.I09	Assess characteristics or impacts of regulations or policies.
4	4.A.2.a.4.I10	Analyze biological or chemical substances or related data.
4	4.A.2.a.4.I11	Analyze business or financial data.
4	4.A.2.a.4.I12	Analyze performance of systems or equipment.
4	4.A.2.b.1.I03	Authorize business activities or transactions.
4	4.A.2.b.1.I08	Determine resource needs of projects or operations.
4	4.A.2.b.1.I09	Implement procedures or processes.
4	4.A.2.b.2.I02	Design computer or information systems or applications.
4	4.A.2.b.2.I03	Develop business or marketing plans.
4	4.A.2.b.2.I06	Design databases.
4	4.A.2.b.2.I07	Develop technical specifications for products or operations.
4	4.A.2.b.2.I08	Develop plans for managing or preserving natural resources.
4	4.A.2.b.2.I09	Develop financial or business plans.
4	4.A.2.b.2.I11	Design structures or facilities.
4	4.A.2.b.2.I12	Develop marketing or promotional materials.
4	4.A.2.b.2.I16	Develop systems or practices to mitigate or resolve environmental problems.

4	4.A.2.b.2.I18	Create visual designs or displays.
4	4.A.2.b.2.I19	Develop contingency or emergency response plans.
4	4.A.2.b.2.I20	Develop sustainable organizational or business policies or practices.
4	4.A.2.b.2.I21	Develop models of systems, processes, or products.
4	4.A.2.b.2.I23	Develop research plans or methodologies.
4	4.A.2.b.2.I24	Design electrical or electronic systems or equipment.
4	4.A.2.b.2.I26	Develop scientific or mathematical theories or models.
4	4.A.2.b.3.I01	Maintain current knowledge in area of expertise.
4	4.A.2.b.4.I01	Develop organizational policies, systems, or processes.
4	4.A.2.b.4.I03	Develop operational or technical procedures or standards.
4	4.A.3.a.3.I09	Operate laboratory or field equipment.
4	4.A.3.b.1.I01	Program computer systems or production equipment.
4	4.A.3.b.1.I02	Implement security measures for computer or information systems.
4	4.A.3.b.1.I03	Set up computer systems, networks, or other information systems.
4	4.A.3.b.1.I04	Resolve computer problems.
4	4.A.3.b.4.I04	Maintain electronic, computer, or other technical equipment.
4	4.A.3.b.6.I01	Prepare financial documents, reports, or budgets.
4	4.A.3.b.6.I03	Present research or technical information.
4	4.A.3.b.6.I07	Prepare proposals or grant applications.
4	4.A.3.b.6.I09	Document technical designs, procedures, or activities.
4	4.A.3.b.6.I14	Prepare legal or regulatory documents.
4	4.A.3.b.6.I15	Prepare reports of operational or procedural activities.
4	4.A.4.a.2.I01	Communicate environmental or sustainability information.
4	4.A.4.a.2.I04	Coordinate with others to resolve problems.
4	4.A.4.a.2.I06	Communicate with others about business strategies.
4	4.A.4.a.2.I09	Communicate with others about specifications or project details.
4	4.A.4.a.2.I11	Coordinate activities with clients, agencies, or organizations.
4	4.A.4.a.2.I13	Confer with clients to determine needs or order specifications.
4	4.A.4.a.4.I01	Develop professional relationships or networks.
4	4.A.4.a.7.I02	Negotiate contracts or agreements.
4	4.A.4.a.7.I03	Resolve personnel or operational problems.
4	4.A.4.b.3.I05	Train others to use equipment or products.
4	4.A.4.b.4.I01	Supervise personnel activities.
4	4.A.4.b.4.I06	Direct scientific or technical activities.
4	4.A.4.b.4.I07	Manage human resources activities.
4	4.A.4.b.4.I08	Manage control systems or activities.
4	4.A.4.b.4.I09	Manage budgets or finances.
4	4.A.4.b.4.I10	Direct construction or extraction activities.
4	4.A.4.b.4.I14	Coordinate regulatory compliance activities.
4	4.A.4.b.6.I03	Advise others on environmental sustainability or green practices.
4	4.A.4.b.6.I04	Advise others on the design or use of technologies.
4	4.A.4.b.6.I05	Advise others on business or operational matters.
4	4.A.4.b.6.I08	Advise others on legal or regulatory matters.
4	4.A.4.b.6.I11	Advise others on financial matters.
4	4.A.4.c.2.I01	Perform recruiting or hiring activities.

S5. Further statistical analysis

The correlation between skills tightness and GDP per capita remain positive and significant when controlling for MSA population. As correlations are reported in the paper, regressions analysis of GDP Per Capita are reported in Table S0-1.

Table S5-1. Regressions Explaining MSA GDP Per Capita 2018

Variables	(1) Element	(2) Element (Z-Score)	(3) Element (Z-Score)	(4) IWA	(5) IWA (Z-Score)	(6) IWA (Z-Score)
Tightness	1619.75***	6.09***	4.74***	7215.51***	6.70***	5.73***
	(191.11)	(0.72)	(0.81)	(752.95)	(0.70)	(0.71)
Population (Log)	-	-	2.63***	-	-	3.24***
	-	-	(0.75)	-	-	(0.66)
Constant	43.08***	48.03***	14.67	42.85***	47.95***	6.79
	(0.93)	(0.72)	(9.56)	(0.89)	(0.71)	(8.43)
Observations	382	382	382	382	382	382
Adj. R^2	0.16	0.16	0.18	0.19	0.19	0.24
Note: Standard errors are shown in parentheses, ***p < 0.01, **p < 0.05, *p < 0.1.						

Supplemental References

1. Blondel VD, Guillaume J-L, Lambiotte R, Lefebvre E. Fast unfolding of communities in large networks. Journal of Statistical Mechanics: Theory and Experiment. 2008; 2008(10):P10008.
2. de Nooy W, Mrvar A, Batagelj V. Exploratory Social Network Analysis with Pajek, 2nd Ed. Granovetter M, editor. New York, NY: Cambridge University Press; 2011.
3. National Center for O*NET Development. O*NET OnLine. Available at: https://www.onetonline.org/; 2020
4. U.S. Bureau of Labor Statistics. Occupational Employment Survey. Available at: http://www.bls.gov/oes/; 2018
5. Office of Management and Budget. OMB Bulletin No. 20-01: Revised Delineations of Metropolitan Statistical Areas, Micropolitan Statistical Areas, and Combined Statistical Areas, and Guidance on Uses of the Delineations of These Areas. Washington, DC; 2020.
